# Combination of copanlisib with cetuximab improves tumor response in cetuximab-resistant patient-derived xenografts of head and neck cancer

**DOI:** 10.18632/oncotarget.27763

**Published:** 2020-10-13

**Authors:** Konrad Klinghammer, Oliver Politz, Theresa Eder, Raik Otto, Jan-Dirk Raguse, Andreas Albers, Andreas Kaufmann, Ingeborg Tinhofer, Jens Hoffmann, Ulrich Keller, Ulrich Keilholz

**Affiliations:** ^1^Department of Hematology and Medical Oncology, Charité, Berlin, Germany; ^2^Bayer AG, Research & Development, Pharmaceuticals, Berlin, Germany; ^3^Department of Radiooncology and Radiotherapy, Charité University Hospital, Berlin, Germany; ^4^German Cancer Research Center (DKFZ), Heidelberg and German Cancer Consortium (DKTK) Partner Sites, Berlin, Germany; ^5^WBI, Humboldt-Universität zu Berlin, Berlin, Germany; ^6^Department of Maxillio-Facial Surgery, Fachklinik Hornheide, Münster, Germany; ^7^Department of ENT, Charité, Berlin, Germany; ^8^Department of Gynaecology, Charité, Berlin, Germany; ^9^Experimental Pharmacology & Oncology GmbH, Berlin, Germany; ^10^Charité Comprehensive Cancer Center, Berlin, Germany

**Keywords:** cetuximab, copanlisib, head and neck squamous cell carcinoma, HPV, patient-derived xenograft model

## Abstract

Despite recent advances, the treatment of head and neck squamous cell carcinoma (HNSCC) remains an area of high unmet medical need. HNSCC is frequently associated with either amplification or mutational changes in the PI3K pathway, making PI3K an attractive target particularly in cetuximab-resistant tumors. Here, we explored the antitumor activity of the selective, pan-class I PI3K inhibitor copanlisib with predominant activity towards PI3Kα and δ in monotherapy and in combination with cetuximab using a mouse clinical trial set-up with 33 patient-derived xenograft (PDX) models with known HPV and PI3K mutational status and available data on cetuximab sensitivity. Treatment with copanlisib alone resulted in moderate antitumor activity with 12/33 PDX models showing either tumor stabilization or regression. Combination treatment with copanlisib and cetuximab was superior to either of the monotherapies alone in the majority of the models (21/33), and the effect was particularly pronounced in cetuximab-resistant tumors (14/16). While no correlation was observed between PI3K mutation status and response to either cetuximab or copanlisib, increased PI3K signaling activity evaluated through gene expression profiling showed a positive correlation with response to copanlisib. Together, these data support further investigation of PI3K inhibition in HNSCC and suggests gene expression patterns associated with PI3K signaling as a potential biomarker for predicting treatment responses.

## INTRODUCTION

Head and neck squamous cell carcinoma (HNSCC) represent the sixth most common type of cancer with 0.65 million new cases and 0.33 million deaths annually worldwide [1]. HNSCC are divided into human papilloma virus (HPV)-positive and HPV-negative carcinomas, the former being associated with better prognosis [2]. Despite recent advances in the diagnosis and treatment of HNSCC, the median survival for patients with incurable, recurrent, or metastatic disease remains poor. To date, the epidermal growth factor receptor (EGFR)-targeted antibody cetuximab and programmed death receptor-1 (PD-1) antibodies nivolumab and pembrolizumab are approved as targeted agents for the treatment of HNSCC. Cetuximab monotherapy has showed modest activity in patients with platinum-refractory HNSCC with a response rate of 13% [3]. Combining cetuximab with chemotherapy demonstrated increased overall survival and progression-free survival in recurrent/metastatic disease [4].

The phosphatidylinositol 3-kinase (PI3K) pathway is a key regulator of cellular physiology, and PI3K/AKT/mTOR signaling is known to enhance tumor cell proliferation, survival and motility in various cancers [5, 6]. Upregulated PI3K/AKT/mTOR signaling has also been shown to increase resistance to radiotherapy and cytostatic drugs [7, 8], and PI3K has been defined as an alternate signaling pathway in cetuximab-resistant HNSCC [9]. Accordingly, based on the cancer genome atlas (TCGA) data, up to 56% of HNSCC display either amplification or mutational changes in the PI3K pathway [10], and according to the head and neck cancer tissue array data, the PI3K/AKT/mTOR pathway is upregulated in over 90% in both HPV-positive and negative HNSCCs [11].

Emerging evidence from preclinical and clinical studies suggest that inhibitors of the PI3K/AKT/mTOR pathway could have potential in combination with other anticancer therapies to circumvent resistance by cancer cells [12]. For instance, PI3K inhibition with buparlisib has shown promising results in patients with recurrent/metastatic HNSCC in combination with paclitaxel [13].

Upregulated PI3K/AKT/mTOR signaling, the occurrence of mutational changes in the PI3K pathway and emerging clinical data from trials with PI3K inhibitors make PI3K an attractive target for cancer therapy, particularly in patients with recurrent/metastatic HNSCC.

Copanlisib is a highly selective, pan-class I PI3K inhibitor with preferential activity against the p110α and p110Δ isoforms that lead to downregulation of PI3K signaling [14]. Copanlisib has been approved for the treatment of follicular lymphoma in the U.S. and demonstrates manageable safety profile in long-term treatment with no late-onset toxicities [15]. Here, we explored the antitumor activity of copanlisib in HNSCC in preclinical trial setup. Due to the ability of mirroring human tumor biology by displaying similar genomic, histopathologic, protein expression and epigenetic tumor features patient-derived xenografts (PDX) have been advocated to predict phase II trial results [16]. To this end, a panel of altogether 33 HPV-positive or negative HNSCC PDX models were selected based on *PI3KCA* mutation status and cetuximab sensitivity to evaluate the efficacy of copanlisib given as monotherapy and in combination with cetuximab, and furthermore, the capacity of copanlisib to overcome resistance to cetuximab. Next to response, potential biomarkers of treatment response such as *PI3KCA* mutation and PI3K pathway activity were evaluated.

## RESULTS

### Establishing and characterizing a panel of PDX models of HNSCC

We established a panel of HPV-positive (*n* = 10) and HPV-negative (*n* = 57) PDX models of HNSCC as described previously [17, 18]. In the present study, a total of thirty-three PDX models of HNSCC were used for evaluation of the *in vivo* efficacy of copanlisib as monotherapy and in combination with cetuximab. Of the thirty-three, six tumors were classified as HPV-positive. Information on patient and tumor characteristics are summarized in Supplementary Table 1.

### *In vivo* efficacy of copanlisib as monotherapy and in combination with cetuximab in PDX models of HNSCC

**Figure 1:**
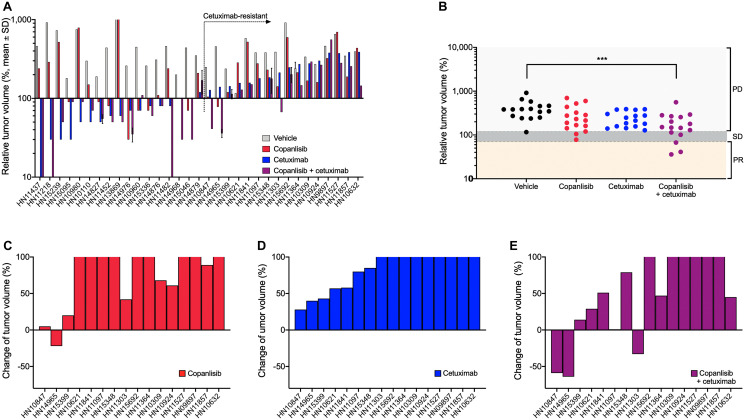
*In vivo* antitumor efficacy of copanlisib and cetuximab in the panel of HNSCC PDX models. (**A**) NMRI *nu/nu* or NOG (for HN15239) mice bearing HNSCC xenografts (*n* = 33) were treated with copanlisib (intravenously at 10 mg/kg, 2on/5off) and/or cetuximab (intravenously at 50 mg/kg, once weekly) for three weeks. (**B**–**E**) Tumor growth in the cetuximab-resistant HNSCC PDX models (*n* = 16) described in panel (A). Relative tumor volume (A–B) and change of tumor volume (C–E) are calculated from the difference of tumor size at the end of the treatment period compared to the initial tumor size (at the time point of starting treatment) of each individual animal. PR, partial response; SD, stable disease; and PD, progressive disease. Asterisks indicate statistical significance, *p* < 0.001.

**Figure 2:**
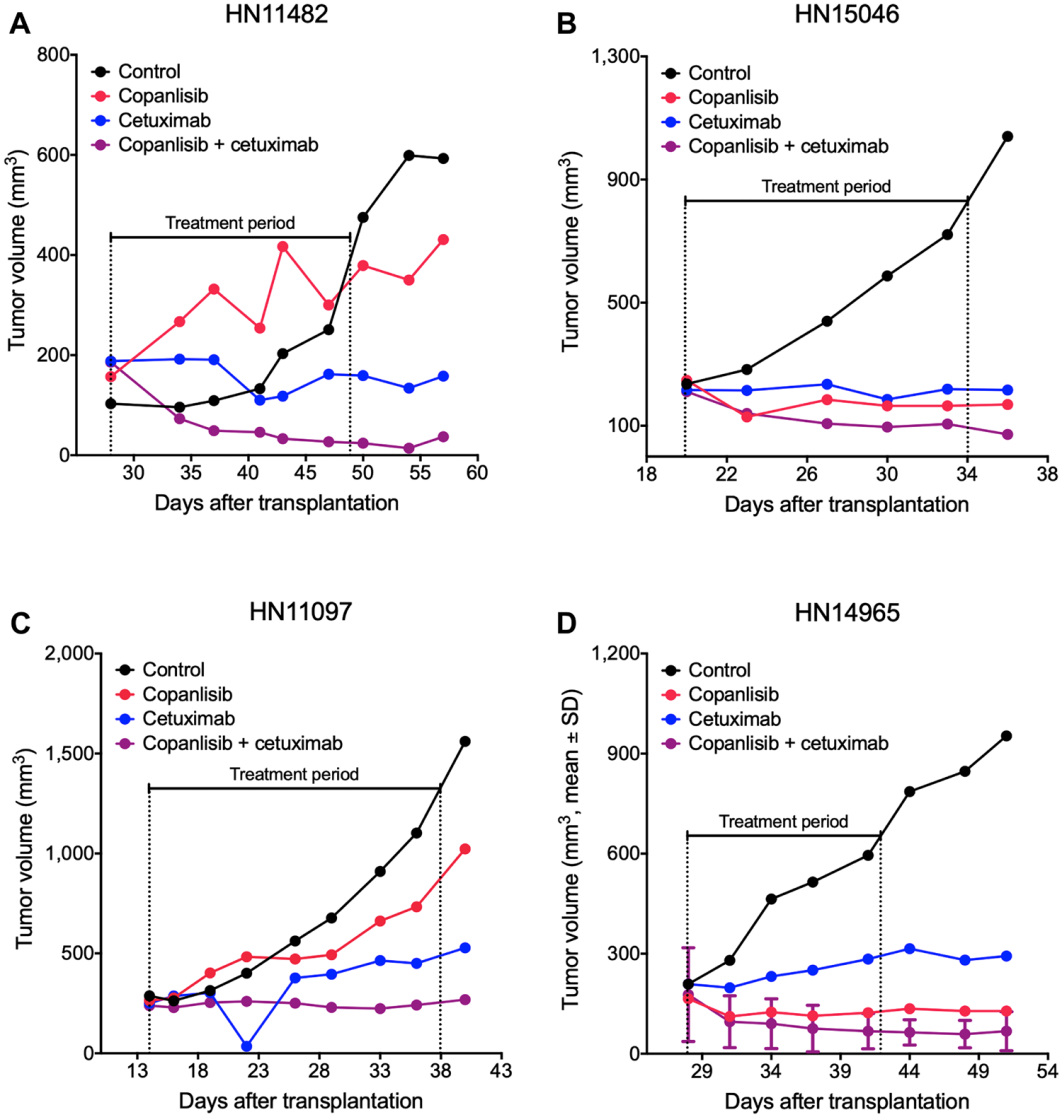
Representative tumor growth curves from the panel of HNSCC PDX models. NMRI *nu/nu* mice bearing cetuximab-sensitive HN11482 (**A**) and HN15046 (**B**) or cetuximab-resistant HN11097 (**C**) and HN14965 (**D**) xenografts were treated with copanlisib (intravenously at 10 mg/kg, 2on/5off) and/or cetuximab (intravenously at 50 mg/kg, once weekly) for three weeks. Tumor volume was measured twice weekly. HN14965 (D) represents an HPV-positive model (*n* = 2).

Antitumor efficacy of copanlisib as monotherapy as well as the combination with cetuximab was assessed in the selected PDX models of HNSCC. Following copanlisib monotherapy, a marked heterogeneity across the PDX models was observed with partial remission or stable disease in 36% (12/33) of the models (Table 1). Approximately half of the models (16/33), including all the HPV-positive PDX models, showed no response to cetuximab monotherapy (relative tumor volume, RTV > 1.2) and were therefore defined as cetuximab-resistant. When combining copanlisib with cetuximab, an improved treatment response was detected in 21 out of 33 models (Figure 1A). For the cetuximab-resistant tumors, i.e., the PDX models that showed no response to cetuximab monotherapy (RTV > 1.2), the treatment response was improved in 88% (14/16) of the models indicated by lower RTV values in the combination group (Table 1; Figure 1B–1E). For the HPV-positive tumors, treatment with copanlisib in combination with cetuximab resulted in an improved efficacy compared to both monotherapies in 83% (5/6) of the models, as determined by lower RTV values (Table 1). Individual growth curves of tumors from representative models are shown in Figure 2.

**Table 1:** Antitumor efficacy of copanlisib and cetuximab in PDX models of HNSCC

	Response to therapy (RTV^b^)		
Tumor ID	Copanlisib	Cetuximab	Copanlisib + cetuximab
HN11437	PD (2.4)	PR (0.1)	PR (0.1)
HN11218	PD (2.9)	PR (0.3)	CR (0.0)
HN15239	PD (5.2)	PR (0.3)	PR (0.5)
HN15095	SD (0.9)	PR (0.3)	SD (0.9)
HN10980	PD (7.9)	PR (0.5)	SD (0.9)
HN10110	PD (1.5)	PR (0.5)	SD (0.7)
HN14827	SD (0.9)	PR (0.5)	PR (0.5)
HN11452	SD (0.8)	PR (0.6)	PR (0.5)
HN13869	PD (13.1)	PR (0.6)	PR (0.5)
HN14976	PR (0.3)	SD (0.7)	PR (0.3)
HN10960	SD (0.7)	SD (0.7)	SD (1.1)
HN15336	SD (0.7)	SD (0.8)	PR (0.6)
HN14876	SD (1.1)	SD (0.8)	SD (0.8)
HN11482	PD (2.4)	SD (0.8)	PR (0.1)
HN14968	SD (1.0)	SD (1.0)	PR (0.3)
HN15046	SD (0.7)	SD (1.0)	PR (0.3)
HN14879	PD (2.1)	SD (1.2)	PD (1.7)
HN10847	SD (1.0)	PD (1.3)	PR (0.4)
HN14965^a^	SD (0.8)	PD (1.4)	PR (0.3)
HN15399^a^	SD (1.2)	PD (1.4)	SD (1.1)
HN10621	PD (2.8)	PD (1.6)	PD (1.3)
HN11841	PD (5.2)	PD (1.6)	PD (1.5)
HN11097	PD (2.8)	PD (1.8)	SD (1.0)
HN15348	PD (2.3)	PD (1.8)	PD (1.7)
HN11303^a^	PD (1.4)	PD (2.1)	SD (0.7)
HN15692^a^	PD (6.0)	PD (2.5)	PD (2.0)
HN11364^a^	PD (2.1)	PD (2.7)	PD (1.5)
HN10309^a^	PD (1.7)	PD (2.8)	PD (2.9)
HN10924	PD (1.6)	PD (3.0)	PD (2.7)
HN9897	PD (3.2)	PD (3.8)	PD (5.6)
HN11527	PD (7.0)	PD (3.8)	PD (2.8)
HN11857	PD (1.9)	PD (3.9)	PD (2.6)
HN10632	PD (4.4)	PD (3.9)	PD (1.4)

RECIST criteria on response to therapy: complete response, CR (RTV < 0.1), partial response, PR (RTV 0.1–0.6), stable disease, SD (RTV 0.7–1.2) and progressive disease, PD (RTV > 1.2). In the cetuximab treatment group, models with RTV > 1.2 were defined as cetuximab-resistant. ^a^HPV-positive models; ^b^RTV, relative tumor volume at the end of the treatment period (3 weeks) in comparison to the timepoint of treatment initiation.

### PI3KCA mutational status and response to copanlisib in PDX models of HNSCC

**Figure 3:**
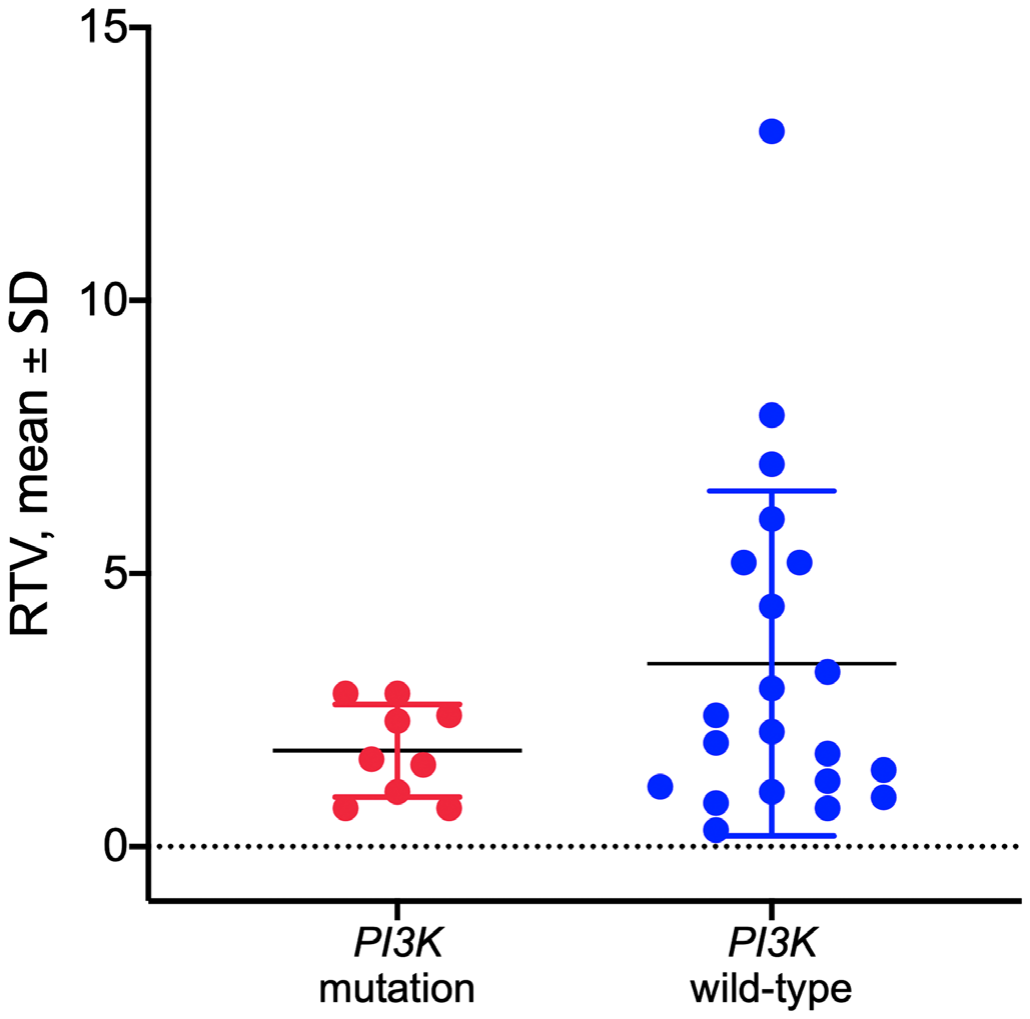
Response to copanlisib expressed as relative tumor volume (RTV) in HNSCC PDX models with mutated or wild-type PI3K gene. No correlation was observed between the *PI3K* mutational status and response to copanlisib (*p* = 0.1516, unpaired *t*-test). Data are expressed as dot plots with each dot representing individual PDX model with mean and SD represented by horizontal lines.

Tumor DNA derived from PDX in the control group was sequenced for *PI3KCA* to determine the mutational status of the PDX models as described previously [18]. Hot spot mutations within the *PI3KCA* gene in the helical or kinase domain were detected in 9 out of 33 established PDX models (Supplementary Table 2). No significant correlation was observed between the *PI3KCA* mutational status and response to copanlisib in the models tested (*p* = 0.1516, unpaired *t*-test; Figure 3).

### PI3K signaling activity and response to copanlisib in PDX models of HNSCC

**Figure 4:**
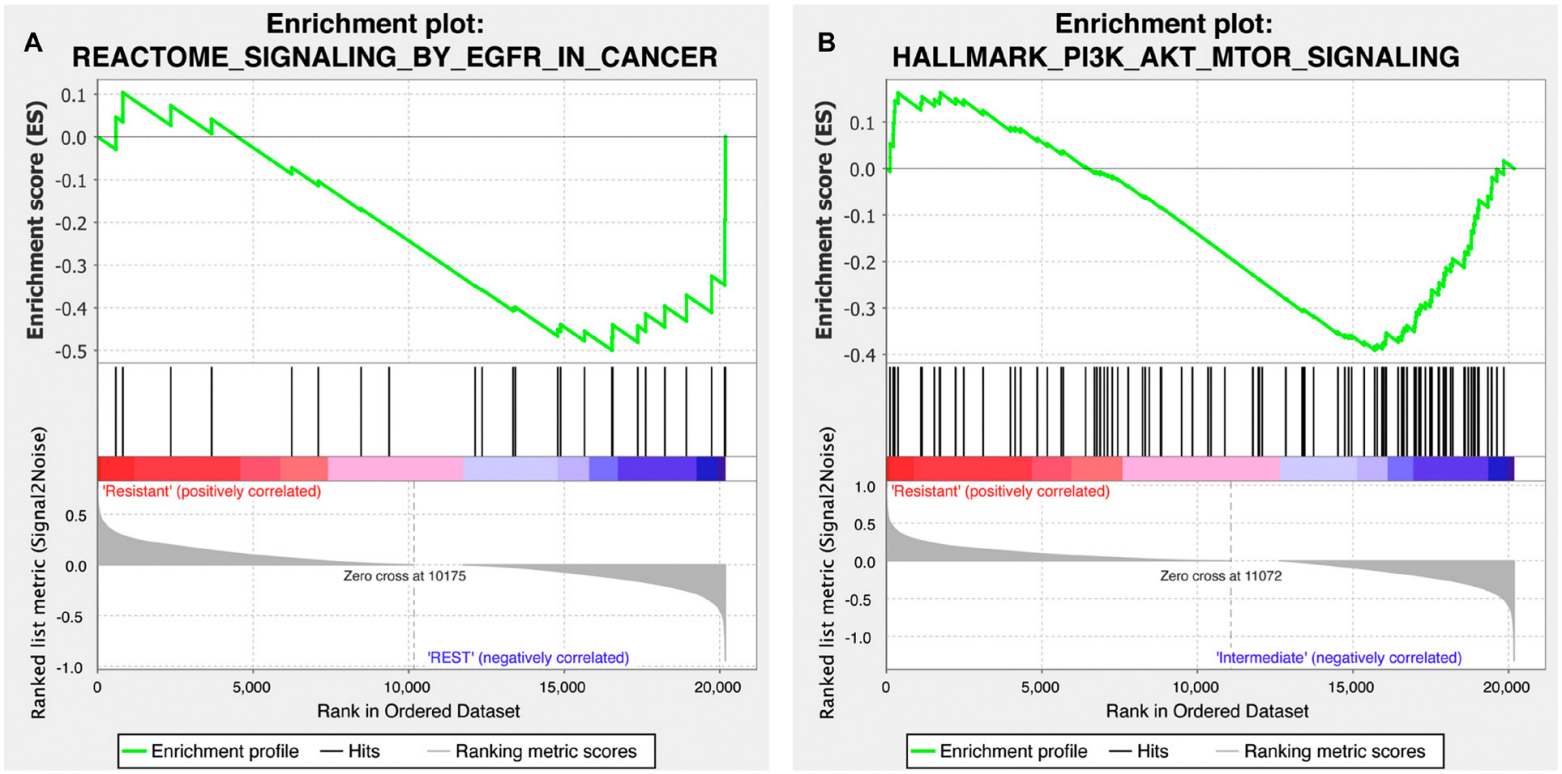
Gene set enrichment plot of the EGFR and PI3K-AKT-mTOR signaling pathways for the copanlisib and cetuximab cohorts. (**A**) Cetuximab-responsive cohort (labeled as ‘REST’ on the plot) presented with a differentially stronger activity of EGFR signaling (MSigDB id M563) compared to the cetuximab-resistant HNSCC samples (*p* = 0.040; false discovery rate, *q* = 0.022). (**B**) Copanlisib-responsive cohort (labeled as ‘Intermediate’ on the plot) was found to harbor a differentially stronger activity of PI3K-AKT-mTOR pathway (MSigDB id M5923) compared to the copanlisib-resistant HNSCC samples (*p* = 0.03; *q* = 0.067).

To gain further insight into the differential response to treatment observed in our PDX models, we evaluated the functional pathway enrichment between response to copanlisib and cetuximab and the expression of genes involved in the PI3K-AKT-mTOR and EGFR pathways employing gene set enrichment analysis (GSEA), respectively. Based on 19 samples for which both drug-response and expression data were available, we found that the EGFR signaling pathway gene set was significantly enriched in the cetuximab-responsive cohort (*p* = 0.040; Figure 4A) while the copanlisib-responsive cohort was significantly enriched for the PI3K-AKT-mTOR hallmark pathway gene set (*p* = 0.03; Figure 4B).

## DISCUSSION

Patient-derived xenografts have attracted a growing attention due to recapitulating patient response in the clinical setting much better than traditional cell line models. This has been mainly attributed to the reflection and maintenance of tumor biology in PDX, proven for various features. For instance, correlation of the mutational profiles between original tumors and PDX models has been shown to be significantly higher than in established tumor cell lines [19]. By employing a one mouse, one tumor, one treatment trial design (1 × 1 × 1), a large number of heterogenous tumors can be evaluated, and a clinical phase II trial can be mimicked in PDX with manageable costs and efforts [20]. This approach has been shown to be feasible and more reliable in predicting the clinical response category (complete response, partial response, stable disease and progressive disease), rather than using a limited number of tumors with a higher number of replicates [19, 21–23]. As a result, comparing drug efficacy with a probability of type I error (a) = 0.05 and a power of 0.8 with a lower proportion for rejection (p0) = 0.1 and a higher proportion for acceptance (pn) = 0.25 becomes already possible with a sample size of 33 [24]. Hence, drug testing in at least 33 PDX models with the common features such as histology or a particular molecular aberration is feasible and can lead to robust information whether or not a drug is active in the chosen setting.

*EGFR* is overexpressed in over 90% of head and neck squamous cell cancers [25]. Cetuximab, which targets the ligand-binding domain of the EGFR, has been the only targeted drug in HNSCC until recently when checkpoint inhibitors were introduced. Cetuximab has, however, low response rates when used as a single agent due to a complex network of alternate signaling of the EGFR pathway. One of the proposed resistance mechanisms of EGFR inhibition is the activation of PI3K signaling [9]. Indeed, *PI3KCA* gene mutation is the most commonly identified targetable genetic aberration found in head and neck cancer and predominantly found in HPV-positive carcinomas. This is of particular relevance since in the U. S. where HPV accounts for 73% of oropharyngeal carcinomas [26]. Further, PI3K inhibition has led to promising results in patients with platinum-pretreated recurrent or metastatic HNSCC in the BERIL-1 phase II trial [27]. Buparlisib, the PI3K inhibitor used in the trial, was not further developed most likely due to its unfavorable toxicity profile in this vulnerable population. The pan-class-I PI3K inhibitor copanlisib, on the other hand, has demonstrated a manageable safety profile in long-term treatment with no late-onset toxicities in lymphoma [15]. This, together with the biological rationale, makes the combination of cetuximab with copanlisib attractive. We observed a limited single agent activity of copanlisib in 11 of 33 models showing tumor growth arrest or tumor regression, which may be translated to a disease control rate of 33%. However, by combining cetuximab and copanlisib we demonstrated improved tumor response in cetuximab-resistant models. This effect was particularly pronounced in HPV-positive models, although, the low number of available HPV models sets limitations to the interpretation of the results.

In contrast to what has been reported for another PI3K-targeting compound alpelisib [28], we did not observe association between the treatment response and *PI3KCA* mutation. This might be due to the limited number of models with mutations in the *PI3KCA* gene. Differing activities against the p110α and p110Δ isoforms by the PI3K compounds may also play a role. On the other hand, copanlisib has also shown activity in *PIK3CA* wild-type models in a preclinical setting [14], While our results argues against *PI3KCA* mutation as predictive biomarker for the stratification of patients, we showed that PI3K pathway activity could potentially be used to identify tumors with higher sensitivity to cetuximab and copanlisib combination therapy. Indeed, the copanlisib-responsive models indicated increased PI3K signaling. In conclusion, we demonstrated an improved tumor control by combining copanlisib with cetuximab using 33 patient-derived xenograft (PDX) models, and thereby establishing a preclinical rationale for the evaluation of this combination therapy in a clinical setting. A phase Ib/II study of copanlisib in combination with cetuximab in HNSCC patients harboring a *PI3KCA* mutation/amplification and/or a *PTEN* loss is currently ongoing (NCT02822482).

## MATERIALS AND METHODS

### Compounds, patient-derived tumors and xenograft models

The pan-class I PI3K inhibitor copanlisib (BAY 841236) was manufactured by Bayer AG (Germany), and the EGFR inhibitor cetuximab was obtained from Merck (Darmstadt, Germany). The vehicle used was 5% mannitol in water for copanlisib and saline (0.9% NaCl) for cetuximab [18].

The PDX models were established at EPO Berlin-Buch GmbH (Germany) and propagated subcutaneously in NMRI *nu/nu* or NOG (for HN15239) mice (Janvier, France) as described previously [18]. EPO is fully accredited by AAALAC. For transplantation, tumors were harvested and cut into small fragments. The study was approved by the local Institutional Review Board of Charité University Medicine, Germany (EA4/019/12). All animal experiments were carried out in accordance with the United Kingdom coordinating committee on cancer research regulations for the welfare of animals and the German Animal Protection Law and were also approved by the local responsible authorities (LaGeSoBerlin, A0452/08).

### *In vivo* efficacy

*In vivo* efficacy of copanlisib as monotherapy and in combination with cetuximab was assessed in 33 PDX models of HNSCC using a mouse clinical trial setting with one mouse per model per arm. For eight models (HN14827, HN14879, HN14965, HN14976, HN15336, HN15348, HN15399, HN15692) in the copanlisib and cetuximab combination group and one model (HN11364) in the vehicle group, two replicates were used. Tumor fragments of similar size (2 × 2 mm) were transplanted subcutaneously to NMRI *nu/nu* mice or NOG (for HN15239). At palpable tumor size of 200 mm^3^, the mice were treated with copanlisib (10 mg/kg, i.v.) twice a week (QD, 2 on/5 off) and/or cetuximab (50 mg/kg, i.v.) once a week (Q7D). Mice in the vehicle group received 5% mannitol in water or 0.9% NaCl. Tumor growth was monitored two times a week by measuring tumor volume [(width^2^ × length)/2] using a caliper. Treatment was continued over a period of 3 weeks unless tumor size exceeded 2,000 mm^3^ or animals showed a loss of body weight over 10%. Relative tumor volume (RTV) was calculated by dividing the tumor volume at the end of the study by the tumor volume at the timepoint of treatment initiation for each individual mouse. Groups of responders were defined based on Response Evaluation Criteria in Solid Tumors (RECIST): complete remission, CR (RTV < 0.1), partial remission, PR (RTV 0.1–0.6), stable disease, SD (RTV 0.7–1.2) and progressive disease, PD (RTV > 1.2).

### Assessment of PI3K mutational status

DNA was extracted using Qiagen DNeasy Extraction kit according to the manufacturer’s protocol. Mutational profiling of PDX models was performed using an in-house gene panel targeting 327 genes (Supplementary Table 3) with the HaloplexHS target enrichment system (Agilent, Santa Clara, CA, USA) as described previously [29]. Sequencing analysis was carried out on the Illumina NextSeq500 platform (Illumina, San Diego, CA, USA). Raw FASTQ files were further processed with Agilent SureCall Software (version 3.5.1.46). A more detailed description of sequencing, data processing and analysis is provided in the Supplementary Data.

### HPV genotyping

HPV genotyping was performed on FFPE material derived from xenografts of two different passages per PDX. DNA was isolated from FFPE sections (5 × 10 μm) using Maxwell FFPE Kit (Promega, Walldorf, Germany) according to the manufacturer’s instructions. Five μL DNA solution was used for PCR amplification by BSGP5+/GP6+ generic primers [30] and analyzed by MPG-Luminex. To exclude any cross-contamination during processing, mouse liver FFPE specimens were sectioned between the experimental PDX FFPE material and analyzed for HPV and human β-globin negativity.

### Network signaling

Tumor RNA derived from a PDX in the control group was extracted and analyzed on gene expression array as described previously [17]. In brief, RNA from the vehicle-treated control tumors was extracted using Qiagen RNeasy Kit according to the manufacturer’s protocol. The integrity of RNA was determined using the Agilent 2100 Bioanalyzer. Total RNA was assayed using Affymetrix Human Genome U133 Plus 2.0 microarrays. Quality control procedures were applied to probe-level intensity files. Expression and drug response data were available for 19 samples. Array data quality control included but was not limited to outlier control, RNA degradation score and MA plot verification. After quality control, the data was normalized with the GCRMA algorithm. The samples were grouped according to their respective response to cetuximab and copanlisib and classified based on RECIST criteria as either resistant (PD), intermediate (SD) or sensitive (PR or CR). Six of the cetuximab cohort samples were marked as resistant, two as intermediate and eleven as resistant. Altogether 16 samples were marked as resistant in the copanlisib cohort while three were marked as intermediate.

GSEA analyses were run with the standard Broad institute GSEA Java software version 4.0.3. The data were normalized using R (version 3.6.1) [31] applying the affyPLM package. The expression data was filtered such that only one probe represented a gene by selecting for the probe with highest variance between the cohorts. The GSEA algorithm was run with 1,000 perturbations and gene set randomization and all other parameters set to standard default values. The significance threshold was defined as a *p*-value of 0.05 and the analyzed data is available online on the Gene Expression Omnibus, GSE84713. (http://www.ncbi.nlm.nih.gov/geo/). The Hallmark all v7.1 HGNC symbols. gmt pathway definition file was utilized. We added four pathways, one of which was the significantly negatively cetuximab-enriched pathway ‘REACTOME_SIGNALING_BY_EGFR_IN_CANCER’ (M563).

### Statistical analyses

For the *PI3KCA* mutational analysis, statistical analyses of the *in vivo* efficacy of copanlisib as monotherapy and in combination with cetuximab were performed using unpaired *t*-test. For the *in vivo* efficacy data, validity of the model assumptions was checked for the fitted statistical model. Analyses were performed using linear models estimated with common variance for all groups. Mean comparisons between the treatment and control groups were performed using the estimated linear model and corrected for family-wise error rate using Tukeys’s method.

## SUPPLEMENTARY MATERIALS



## Abbreviations

CR: complete response
EGFR: epidermal growth factor receptor
GSEA: gene set enrichment analysis
HNSCC: head and neck squamous cell carcinoma
HPV: human papilloma virus
mTOR: mammalian target of rapamycin
PD: progressive disease
PD-1: programmed death receptor-1
PDX: patient-derived xenograft
PI3K: phosphoinositide 3-kinase
*PI3KCA*: phosphatidylinositol-3-kinase catalytic subunit alpha isoform gene
PR: partial response
PTEN: phosphatase and tensin homologue
RTV: relative tumor volume
TCGA: the cancer genome atlas
SD: stable disease
