# Supplementary material for: Combination of copanlisib with cetuximab improves tumor response in cetuximab-resistant patient-derived xenografts of head and neck cancer
Source: Oncotarget. 2020 Oct 13;11(41):3688–97. doi: 10.18632/oncotarget.27763 (PMC7566806; doi:10.18632/oncotarget.27763)
Supplement: Supplementary file 1 [file oncotarget-11-3688-s001.pdf]

Combination of copanlisib with cetuximab improves tumor response in cetuximab-resistant patient-derived xenografts of head and neck cancer

SUPPLEMENTARY MATERIALS

Sequencing analysis

Mutational profiling was performed using an in-house gene panel targeting 327 genes with the HaloplexHS target enrichment system (Agilent, Santa Clara, CA, USA) as described previously [1]. Genes had been selected based on the results from whole-exome sequencing of three independent HNSCC patient cohorts [2, 3] and the COSMIC database [4]. The complete coding sequence of all exons of the 327 genes was covered, resulting in a target region of 1.47 megabase pairs in total. HaloPlexHS library preparation was performed using approximately 100 ng of DNA (Agilent, protocol version F1, July 2015). Briefly, the gDNA was digested and the HaloPlexHS probe library was hybridized in the presence of the indexing primer cassette and the molecular barcode. The biotinylated DNA-probe hybrids were captured by magnetic purification of the samples with streptavidin-coated magnetic beads. Fragmented targets were amplified via PCR at cycling conditions recommended by the manufacturer. Subsequent to the PCR reaction, the final library was purified using AMPure XP magnetic beads (Beckman Coulter, Krefeld, Germany). Pooled libraries from patient samples were used for paired end (PE) sequencing on the Illumina NextSeq500 platform with a High Output v2 sequencing kit, 300 cycles (Illumina, San Diego, CA, USA), producing 150 bp PE reads. A mean read depth of 466-fold (range, 57–2,396) was achieved after duplicate removal in the target region.

Sequencing data analysis

Processing of the raw FASTQ files, sequencing adaptor trimming, sequence alignment, variant calling and duplicate removal was performed with Agilent SureCall Software (version 3.5.1.46). For alignment to the human reference sequence build 38 (hg19), the BWA-MEM algorithm was applied. Variant calling was performed with a cut-off of 0.05 for allele frequencies. Genome annotations for point mutations and short nucleotide insertions as well as deletions were made by using The Cancer-Related Analysis of Variants Toolkit (CRAVAT v5.2.4) [5]. Variants were filtered based on their functional impact predicted by Cancer-Specific High-Throughput Annotation of Somatic Mutations (CHASM) [6] and Variant Effect Scoring Tool (VEST). Only variants with CHASM and VEST composite gene-level P values of < 0.05 were further considered. For removal of germline variants, entries with minor allele frequencies of > 0.05 reported in exome sequencing projects of healthy populations (1000-Genomes; ESP v.6500) were excluded from the analysis.

SUPPLEMENTARY REFERENCES

1. Eder T, Hess AK, Konschak R, Stromberger C, Johrens K, Fleischer V, Hummel M, Balermpas P, von der Grun J, Linge A, Lohaus F, Krause M, Baumann M, et al. Interference of tumour mutational burden with outcome of patients with head and neck cancer treated with definitive chemoradiation: a multicentre retrospective study of the German Cancer Consortium Radiation Oncology Group. Eur J Cancer. 2019; 116:67–76. https://doi.org/10.1016/j.ejca.2019.04.015. [PubMed]
2. Agrawal N, Frederick MJ, Pickering CR, Bettegowda C, Chang K, Li RJ, Fakhry C, Xie TX, Zhang J, Wang J, Zhang N, El-Naggar AK, Jasser SA, et al. Exome sequencing of head and neck squamous cell carcinoma reveals inactivating mutations in NOTCH1. Science. 2011; 333:1154–7. https://doi.org/10.1126/science.1206923. [PubMed]
3. Stransky N, Egloff AM, Tward AD, Kostic AD, Cibulskis K, Sivachenko A, Kryukov GV, Lawrence MS, Sougnez C, McKenna A, Shefler E, Ramos AH, Stojanov P, et al. The mutational landscape of head and neck squamous cell carcinoma. Science. 2011; 333:1157–60. https://doi.org/10.1126/science.1208130. [PubMed]
4. Forbes SA, Bindal N, Bamford S, Cole C, Kok CY, Beare D, Jia M, Shepherd R, Leung K, Menzies A, Teague JW, Campbell PJ, Stratton MR, et al. COSMIC: mining complete cancer genomes in the Catalogue of Somatic Mutations in Cancer. Nucleic Acids Res. 2011; 39:D945–50. https://doi.org/10.1093/nar/gkq929. [PubMed]
5. Masica DL, Douville C, Tokheim C, Bhattacharya R, Kim R, Moad K, Ryan MC, Karchin R. CRAVAT 4: Cancer-Related Analysis of Variants Toolkit. Cancer Res. 2017; 77:e35–e38. https://doi.org/10.1158/0008-5472.CAN-17-0338. [PubMed]

6. Carter H, Chen S, Isik L, Tyekucheva S, Velculescu VE, Kinzler KW, Vogelstein B, Karchin R. Cancer-specific high-throughput annotation of somatic mutations: computational prediction of driver missense mutations. Cancer Res. 2009; 69:6660–7. https://doi.org/10.1158/0008-5472.CAN-09-1133. [PubMed]

Supplementary Table 1: Clinical characteristics of patients for PDX models of HNSCC

Tumor ID	TNM	Stage UICC 7	Grading	Age	Site of tumor origin	Gender	Primary/Recurrent	HPV status
HN09897	T2N2bM0	IVA	G3	58	hypopharynx	male	recurrent	−
HN10110	T2N2cM0	IVA	G2	69	oral cavity	male	primary	−
HN10309	T4N2cM0	IVA	G3	55	oropharynx	male	primary	+
HN10621	T2N2bM0	IVA	G3	61	oropharynx	male	primary	−
HN10632	T2N1M0	III	G3	60	oral cavity	male	primary	−
HN10847	T2N1M0	III	G2	71	oral cavity	female	recurrent	−
HN10924	T3N2cM0	IVA	G2	65	hypopharynx	male	primary	−
HN10960	T2N0M0	II	G2	63	oral cavity	male	primary	−
HN10980	T4bN2bM0	IVB	G2	59	oral cavity	female	primary	−
HN11097	T4aN2bM0	IVA	G2	75	oral cavity	female	primary	−
HN11218	T4N0M0	IVA	G2	68	oral cavity	female	primary	−
HN11303	T3N1M0	IVA	G2	75	oropharynx	male	primary	+
HN11364	T2N2M0	IVA	G2	67	oropharynx	male	primary	+
HN11437	T4bN2cM0	IVB	G2	56	oral cavity	male	primary	−
HN11452	T2N0M0	II	G2	75	oral cavity	male	primary	−
HN11482	T2N2bM0	IVA	G2	61	oral cavity	male	primary	−
HN11527	T2N2bM0	IVA	G2	74	oral cavity	male	primary	−
HN11841	T1N0M0	I	G2	56	oral cavity	female	recurrent	−
HN11857	T4N2M0	IVA	G1	49	oral cavity	male	primary	−
HN13869	T1N2bM0	IVA	G1	71	oral cavity	male	recurrent	−
HN14827	T3N2bM0	IVA	G2	59	oral cavity	female	primary	−
HN14876	T3N2M0	IVA	G2	59	oral cavity	female	primary	−
HN14879	T3N2bM0	IVA	G3	57	oral cavity	female	primary	−
HN14965	T3N2bM0	IVA	G2	73	oropharynx	male	primary	+
HN14968	T3N0M0	III	G3	51	hypopharynx	male	primary	−
HN14976	T4aN2cM0	IVA	G2	74	larynx	male	primary	−
HN15046	T4N3M1	IVC	G2	61	hypopharynx	male	primary[a]	−
HN15095	T3N2M0	IVA	G2	59	oropharynx	female	primary	−
HN15239	T1N0M0	I	G2	26	oral cavity	female	recurrent	−
HN15336	T3N0M0	III	G2	54	larynx	female	recurrent	−
HN15348	T4N0M0	IVA	G2	54	larynx	male	recurrent	−
HN15399	T4N2cM0	IVA	G2	74	oropharynx	male	primary	+
HN15692	T2N2bM0	IVA	G2	57	oropharynx	male	primary	+

[a]primary metastatic.

Supplementary Table 2: Detected mutations within the *PI3KCA* gene in the panel of HNSCC PDX models

Tumor ID	*PI3K* mutation
HN10110	H1047R
HN10621	E545K
HN10847	G1049R
HN10924	E542Q
HN10960	E542K
HN11097	E542K
HN11482	E545K
HN15046	E545K
HN15348	E453K

Supplementary Table 3: List of genes included in in-house 327-gene panel

Gene panel						
ACACA	CDK4	FANCD2	KDR	NCOR1	PRKDC	SPTBN1
ACTC1	CDKN2A	FANCE	KEAP1	NCOR2	PRMT5	SRC
ADCY2	CHD1L	FANCF	KIT	NCR1	PROX1	STEAP4
AFF1	CLDN18	FANCG	KMT2A	NECAB1	PRSS1	STK11
AGTR1	CNTNAP5	FANCI	KMT2C	NEUROD6	PRUNE2	SULF1
AJUBA	COL11A1	FANCL	KMT2D	NF2	PTEN	SYNCRIP
AKAP12	COL1A2	FANCM	KRAS	NFE2L2	PTPN11	SYNE1
AKT1	CPXCR1	FAT1	KRT14	NOTCH1	PTPRA	SYNE2
AKT2	CRABP2	FBXW7	KRT5	NOTCH2	PTPRC	SYNE3
ALB	CREBBP	FCRL4	LAMA1	NOTCH3	PTPRD	TARP
ALDH1L1	CSMD3	FGFR1	LAMA2	NPY5R	PTPRF	TEK
ALK	CTCF	FGFR2	LAMA3	NRAS	PTPRT	TERF1
ANKRD30B	CTNNB1	FGFR3	LAMA4	NSD1	PTPRZ1	TERF2
ANO1	CUL3	FIGN	LAMA5	NTM	RAC1	TERT
APC	CUX1	FLG	LBP	NUP214	RAD51C	TG
APOB	DACH1	FLT3	LCP1	OR2J2	RAP1A	TGFBR2
AR	DCC	FN1	LILRB1	OR2L13	RAP1B	THBS1
ARHGAP35	DICER1	FOSL2	LIMK1	OR2M2	RASA1	TLN1
ARID1A	DMD	FOXO3	LIN28B	OR2T12	RB1	TLR4
ASAP1	DNM2	GABRB3	LINGO2	OR4C11	RB1CC1	TP53
ASNS	DNMT3A	GRID2	LRFN5	OR4M2	RBM5	TP63
ASXL3	DOCK1	GRM1	LRP1	OR52E2	RECQL4	TPO
ATM	DOK6	GSTM2	LRP1B	OR56A1	REG1A	TPP1
ATR	DPP10	HDAC6	LRP6	OR5D13	RELN	TRIM24
ATRX	DPPA4	HERC2	LRRC4C	PABPC5	RET	TRIM58
AURKC	DSP	HFM1	LRRK2	PALB2	RGS17	TRRAP
AXIN2	EGFR	HIST1H1B	LTBP1	PAPPA2	RHOA	TTF1
B2M	EIF3A	HIST1H2BD	MACC1	PBRM1	ROS1	UBE3A
BCL11A	EIF4G1	HIST1H2BK	MAGEL2	PCDH10	RUNX1T1	UBR5
BCL9	EP300	HIST1H4E	MAP2	PCDH11X	RXRA	UGT2B7
BECN1	EPAS1	HNRNPA2B1	MAP3K7	PCDH15	SDHA	USP9X
BIRC6	EPB41L3	HOXD10	MAPK1	PDGFRA	SELP	VCAN
BLM	EPHA2	HRAS	MAPK9	PEG3	SEMA5A	WRN
BRAF	EPHA3	HSPG2	MBD1	PEX11A	SERPINB4	XPO1
BRCA2	EPHA7	HUWE1	MED1	PFKP	SETBP1	ZBTB16
BRIP1	ERBB2	IFNGR1	MED12	PIK3CA	SIN3A	ZIC1
BRWD3	ERBB4	IGF1R	MET	PIK3CG	SIRT1	ZNF737
C6	ETV6	IL1RAPL1	MME	PIK3R1	SIRT3	ZNF750
C9ORF135	EYA1	IL6ST	MS4A14	PIWIL1	SLC26A7	ZNF804B
CASP8	EZH2	INPP5D	MTOR	PKD1	SLC9A1	
CCND1	F13B	INPPL1	MUC5B	PKN1	SLIT2	
CCNE1	F5	ITGB1	MUC6	PLCB1	SLITRK4	
CD163	FAM101A	JAK1	MYC	PLEC	SLX4	
CD1E	FAM155A	JAK2	MYH1	PLSCR4	SMAD4	
CDH1	FAM222B	JAK3	MYH10	POM121L12	SMARCA2	
CDH10	FANCA	KCNT2	MYH11	POT1	SMARCA4	
CDH11	FANCB	KDM5B	MYH9	PRDX4	SMO	
CDH5	FANCC	KDM6A	NBN	PRIM2	SORCS1	
